# Clinical significance of cerebrovascular complications in patients with acute infective endocarditis: a retrospective analysis of a 12-year single-center experience

**DOI:** 10.1186/1471-2377-14-30

**Published:** 2014-02-15

**Authors:** Seung-Jae Lee, Sam-Sae Oh, Dal-Soo Lim, Chan-Young Na, Jae-Hyun Kim

**Affiliations:** 1 Department of Neurology, Sejong General Hospital, Bucheon, South Korea; 2 Department of Thoracic and Cardiovascular Surgery, Sejong General Hospital, Bucheon, South Korea; 3 Department of Cardiology, Sejong General Hospital, Bucheon, South Korea; 4 Department of Thoracic and Cardiovascular Surgery, Dong-san Medical Center, Keimyung University, Daegu, South Korea

**Keywords:** Infective endocarditis, Cerebrovascular complication, Stroke

## Abstract

**Background:**

Cerebrovascular complications (CVCs) frequently occur in patients with acute infective endocarditis (IE). The aim of this study is to describe the clinical findings of CVCs and to evaluate the impact of CVCs on long-term mortality in patients with IE.

**Methods:**

We retrospectively analyzed 144 patients who fulfilled the modified Duke’s criteria for definite left-sided IE. CVCs were classified into minor (silent cerebral embolism, TIA and stroke with an initial modified Rankin scale ≤ 2) or major (an initial modified Rankin scale ≥ 3) CVCs. Cox proportional hazards model was used for mortality analysis. Hazard ratio (HR) and 95% confidence interval (CI) were obtained.

**Results:**

The mean age of the 144 patients (96 males and 48 females) was 49.1 years (range 6-85 years). A CVC was found in 37 (25.7%) patients. Of these, 25 were treated with surgical therapy. The patients who underwent early surgery within 2 weeks after stroke had a statistical trend toward a higher risk of postoperative brain hemorrhage (50% versus 4.8%, *P* = 0.057 by Fisher exact test). The minor CVC group had a similar risk of death as the no-CVC group (*P* = 0.803; HR 0.856; CI 0.253-2.894), whereas the major CVC group had a higher mortality (*P* = 0.013; HR 2.865; CI 1.254-6.548) than the no-CVC group. In the multivariate analysis, major CVC (*P* = 0.002; HR 3.893; CI 1.649-9.194) was a significant predictor of mortality in IE patients, together with advanced age (*P* = 0.005; HR 3.138; CI 1.421-6.930) and prosthetic valve IE (*P* = 0.008; HR 2.819; CI 1.315-6.044).

**Conclusions:**

IE can give rise to various forms of CVC, most frequently, acute ischemic brain lesions. In our study, major CVC was associated with high risk of mortality although total CVC was not significantly related to the risk of death in patients with IE.

## Background

Cerebrovascular complications (CVCs) frequently occur in patients who are in the active stage of infective endocarditis (IE), and result from cerebral septic embolization of an endocardial vegetation. They include stroke, transient ischemic attack (TIA) and silent cerebral embolism (SCE).

A CVC is generally accepted as a predictor of poor prognosis with an increased mortality in patients with IE [1-3]. Especially, several studies have recently reported that the mortality in IE patients depends on type or severity of IE-related CVC [4,5].

In this study, we attempted to (1) describe the incidence, lesion type and neurologic outcome of CVCs, (2) identify the variables determining the occurrence of CVCs and (3) elucidate the impact of types of CVCs on long-term mortality in patients with IE.

## Methods

Using the endocarditis registry of Sejong Cardiovascular Center, the authors of this study ascertained the names and registry numbers of 282 consecutive patients with suspected IE, who were admitted to the Cardiovascular Center at Sejong General Hospital between January 2000 and September 2012. We then retrospectively reviewed their medical records. From these 282 patients, 104 patients with right-sided IE, 27 with possible left-sided IE, and 7 with incomplete study were excluded. Patients with both left- and right-sided IE were classified into the left-sided group. Finally, we analyzed 144 patients who fulfilled the modified Duke’s criteria [6] for definite left-sided IE, and investigated detailed clinical information including medical history (age, sex, hypertension, diabetes mellitus, Charlson comorbidity index [7], atrial fibrillation, current smoking status, congestive heart failure (CHF) and history of IE), operation records, computed tomography (CT), magnetic resonance imaging (MRI), echocardiography and clinical outcome including mortality. The follow-up data were obtained from outpatient medical records. The study protocol was reviewed and approved by the Institutional Review Board of Sejong General Hospital. In addition, we had consent from patients or their legal guardians to publish clinical details.

Transthoracic and transesophageal echocardiography were performed in all cases. Echocardiographic data included IE-related valve regurgitation, vegetation length, mobility and location. The vegetation length was measured in various planes during the first echocardiography and follow-up studies. It was determined whether the maximal vegetation length was > 1 cm.

Diagnosis of a CVC was based on clinical findings, CT or MRI. CVCs included stroke (ischemic or hemorrhagic), TIA, and SCE. An ischemic stroke was defined as a focal neurologic deficit of an abrupt onset lasting > 24 hours with an evidence of new lesions on brain imaging, whereas TIA was defined as a focal neurologic symptom lasting < 24 hours with or without brain lesions. Hemorrhagic stroke was defined as a neurologic symptom with the presence of intracranial bleeding on CT or MRI, and included primary intracerebral hemorrhage (ICH), hemorrhagic infarct (HI) and subarachnoid hemorrhage (SAH). A new asymptomatic brain lesion with high signal intensity on diffusion MRI was considered as SCE.

CVCs were classified into minor or major CVCs. A minor CVC was diagnosed when the initial neurologic signs were absent, transient or mild. This category included SCEs, TIAs and strokes with an initial modified Rankin scale (mRS) ≤ 2. A major CVC was defined as a stroke that initially caused a moderate to severe disability (mRS ≥ 3). In addition, cerebral infarct combined with intracranial hemorrhage or one type of CVCs with additional neurologic complications (seizure, meningitis or mycotic aneurysm) was considered as a complicated stroke.

The topography of brain lesions was determined using the commonly accepted arterial supply templates for the territorial and border zone areas, as described previously [8-10]. Multiple infarcts were defined as more than two lesions that were topographically distinct (separated in space or discrete on contiguous slices) [11]. An uninterrupted lesion visible in contiguous territories was considered a single lesion.

**Figure 1:**
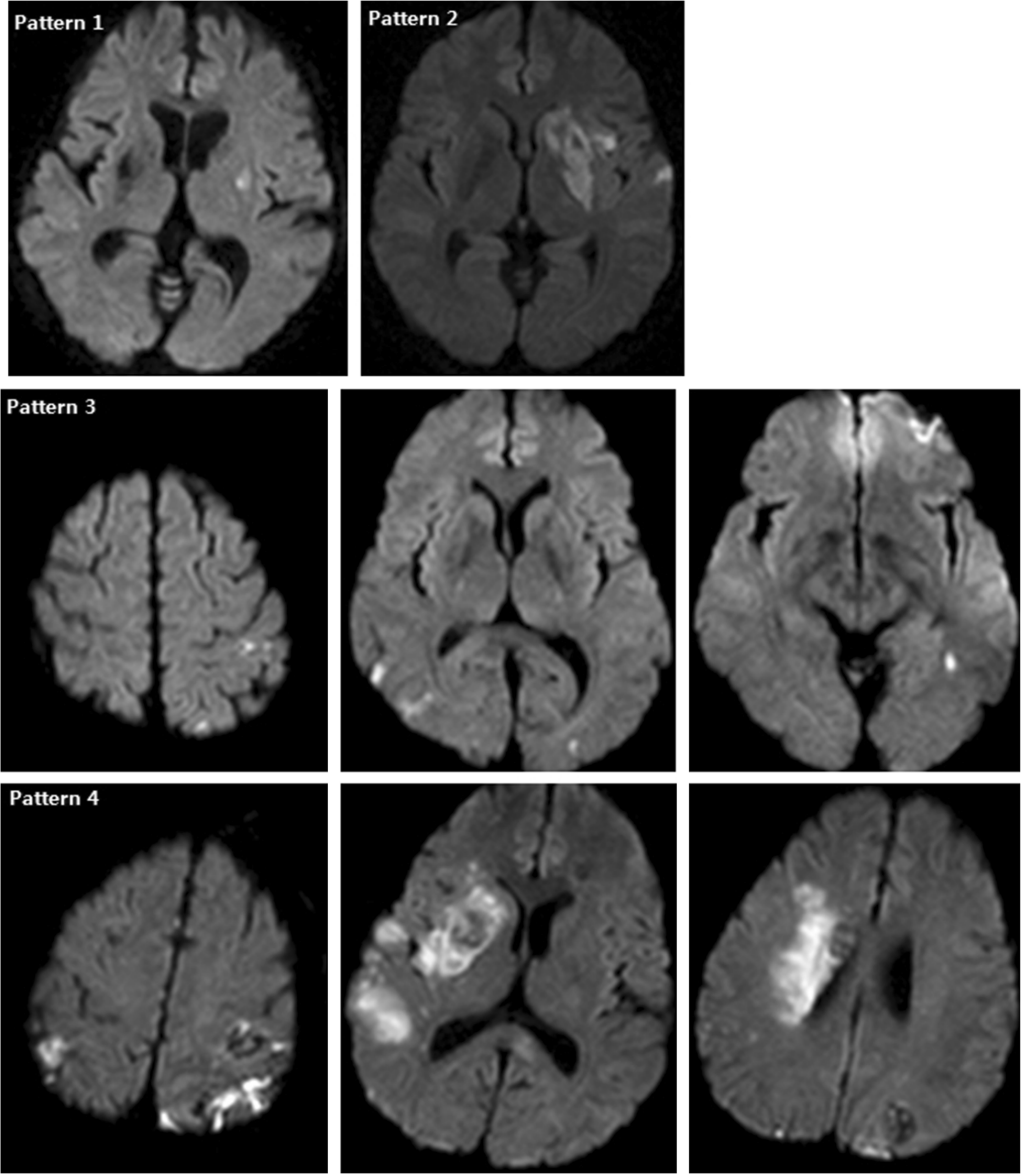
**Patterns of acute embolic lesions on diffusion-weighted MRI in patients with infective endocarditis.** Pattern 1 shown in a 63-year-old female with dysarthria and right hemiparesis, pattern 2 in a 43-year-old male with dysarthria and right hemiparesis, pattern 3 in a 59-year-old female with dysarthria alone, and pattern 4 in a 47-year-old male with confused mentality, dysarthria and left hemiplegia.

As described in a previous study [12], we classified the embolic brain lesions related to IE into the following 4 patterns: (1) single lesion, (2) multiple closely spaced lesions in a single arterial territory- “territorial infarct”, (3) multiple punctate disseminated lesions, and (4) multiple small (< 10 mm) and medium (10-30 mm) or large (> 30 mm) disseminated lesions (Figure 1).

Statistical analyses were performed with SPSS software, version 18.0 (SPSS Inc., Chicago, IL). Independent *t*-test or Chi-square test (or Fisher exact test) was used for comparing the different groups. Multivariate logistic regression analysis was performed to determine the independent predictors for IE-related CVCs. Odds ratio (OR) and 95% confidence interval (CI) were obtained. Besides, the Kaplan-Meier survival curves were computed according to the type of CVC, and compared using the log-rank test. Cox proportional hazards model was used to perform univariate and multivariate analyses for long-term mortality. Unadjusted and adjusted hazards ratio (HR) and CI were obtained. *P-*values < 0.05 were considered statistically significant.

## Results

### General characteristics of the study population

The mean age of 144 patients (96 males and 48 females) included in this study was 49.1 years (range 6-85 years) at admission. There were 134 patients with only left-sided IE and 10 with both-sided IE.

General characteristics of the study patients are shown in Table 1. Streptococci including viridans species were the most common micro-organisms (42 patients, 29.2%). 77 patients (53.5%) underwent brain imaging (CT or MRI) and 37 (25.7%) were confirmed as having acute brain lesions that were comparable to CVCs. Fourteen of 37 patients with CVCs underwent only CT, while the other 23 patients underwent MRI including diffusion-weighted imaging with or without CT.

**Table 1:** General characteristics in patients with and without cerebrovascular complications related to infective endocarditis: mean ± SD, n (%)

	**CVC (-) N = 107**	**CVC (+) N = 37**	***P *** **value**
Age	49.5 ± 18.1	48.0 ± 17.1	0.663
Male	69 (64.5)	27 (73.0%)	0.345
Hypertension	18 (16.8)	7 (18.9)	0.772
Diabetes mellitus	21 (19.6)	6 (16.2)	0.647
Atrial fibrillation	31 (29.0)	12 (32.4)	0.692
Smoking	12 (11.2)	8 (21.6)	0.115
History of IE	1 (0.9)	1 (2.7)	0.449
Dialysis	0 (0.0)	2 (5.4)	0.065
History of CHF	32 (29.9)	9 (24.3)	0.517
Comorbidity index >2	22 (20.6)	9 (24.3)	0.631
Initial C-reactive protein level, mg/dL	7.4 ± 7.3	8.9 ± 6.3	0.437
Diagnostic delay, days	32.8 ± 35.3	29.9 ± 33.3	0.654
Prosthetic valves	45 (42.1)	16 (43.2)	0.900
Mitral valve IE	78 (72.9)	31 (83.8)	0.183
Aortic valve IE	52 (48.6)	17 (45.9)	0.781
Dual valve IE	22 (20.6)	11 (29.7)	0.253
Warfarin use at admission	38 (35.5)	11 (29.7)	0.522
No surgery	21 (19.6)	12 (32.4)	0.111
Causative microorganism			
Streptococcus viridans	18 (16.8)	4 (10.8)	0.441
Other streptococci	15 (14.0)	5 (13.5)	0.939
Staphylococcus aureus	10 (9.3)	9 (24.3)	0.020
Enterococci	6 (5.6)	2 (5.4)	1.000
Coagulase negative staphylococci	4 (3.7)	2 (5.4)	0.647
Others	30 (28.0)	7 (18.9)	0.274
Negative blood cultures	24 (22.4)	8 (21.6)	0.919
Echocardiographic findings			
Vegetations	99 (92.5)	37 (100)	0.113
Vegetation size >1 cm	46 (43.0)	24 (64.9)	0.022
Mobile vegetations	43 (40.2)	29 (78.4)	<0.001
IE-related valve regurgitation	88 (82.2)	30 (81.1)	0.874
In-hospital mortality	18 (16.8)	10 (27.0)	0.176

In addition, 31 peripheral embolic events were identified, involving the spleen in 10 patients (6.9%), kidneys in 7 patients (4.9%), lower limbs in 7 patients (4.9%), heart in 4 patients (2.8%; left anterior descending artery in 3 and right coronary artery in 1) and lungs in 3 patients (2.1%) with both-sided IE.

When compared with the no-CVC group, the CVC group had a significantly higher prevalence of staphylococcus (S.) aureus infection, large (> 1 cm) and mobile vegetation (*P* < 0.05); and showed a statistical trend toward a higher frequency of smoking, dialysis, mitral valve involvement, no surgery and in-hospital mortality (*P* < 0.2). However, there was no significant difference in age, gender, hypertension, diabetes mellitus, atrial fibrillation, history of CHF, comorbidity, C-reactive protein level at admission, period from the initial symptom to diagnosis and warfarin use at admission between the two groups.

Logistic regression analysis (using the variables of mitral valve IE, smoking, S. aureus and mobile vegetation) demonstrated that S. aureus infection (*P* = 0.047; OR 3.012; CI 1.013-8.962) and mobile vegetation (*P* = 0.001; OR 4.768; CI 1.952-11.647) were independent predictors of CVC in patients with left-sided IE.

One hundred and eleven of the 144 patients (77.1%) underwent surgical therapy (53 biologic valves, 36 mechanical valves, 16 valve repairs and 6 aortic homograft valves). When compared with the surgery group, the no-surgery group had a significantly higher proportion of the elderly aged 65 years and older (39.4% versus 14.4%; *P* = 0.002) and high-comorbidity (index > 2) patients (36.4% versus 17.1%; *P* = 0.018).

### Characteristics of the patients with a CVC

Among the 37 patients with a CVC, 30 (81.1%) had an acute ischemic stroke, and 2 (5.4%) had a TIA with a lesion. Besides, only one patient had a SCE. Sixteen (43.2%) of 37 patients with a CVC had hemorrhagic strokes, which included 4 cases of only ICH, 3 cases of ICH with ischemic stroke, 2 cases of SAH with ischemic stroke and 7 cases of HI. In addition, meningitis and mycotic aneurysm were found in one patient each, and 3 patients presented with generalized seizure associated with strokes (Table 2).

**Table 2:** Prevalence of neurologic complications in 144 patients with acute infective endocarditis: n (%)

Total cerebrovascular complications	37 (25.7)
Ischemic stroke	30 (20.8)
TIA	2 (1.4)
SCE	1 (0.7)
Hemorrhagic stroke	16 (11.1)
ICH	7 (4.9)
ICH only	4 (2.8)
ICH + ischemic stroke	3 (2.1)
SAH	2 (1.4)
SAH only	0 (0)
SAH + ischemic stroke	2 (1.4)
HI	7 (4.9)
Meningitis	1 (0.7)
Mycotic aneurysm	1 (0.7)
Seizure	3 (2.1)

TIA, transient ischemic attack; SCE, silent cerebral embolism; ICH, intracerebral hemorrhage; SAH, subarachnoid hemorrhage; HI, hemorrhagic infarct.

Table 3 shows the vascular territories and lesion patterns in 33 patients with ischemic brain lesions. The MCA territory was involved in 23 (69.7%) patients, and it was the most frequently affected arterial territory in patients with IE. Among these patients, most (19 patients, 82.6%) had a partial middle cerebral artery (MCA) stroke, and only 4 (17.4%) presented with a complete MCA stroke. Posterior cerebral artery and posterior inferior cerebellar artery territories were the second most frequently involved regions (24.2%), followed by posterior border zone (15.2%), anterior cerebral artery (12.1%), basilar artery (9.1%), internal border zone (9.1%), superior cerebellar artery (6.1%) and anterior inferior cerebellar artery (3.0%) territories. The patients with IE had various patterns of lesions, from pattern 1 to 4. Most of the lesions were multiple (84.8%); and territorial (pattern 2, 33%) and disseminated small and large lesions (pattern 4, 30.3%) were the prominent lesion patterns.

**Table 3:** Imaging characteristics of 33 patients with ischemic brain lesions related to infective endocarditis: n (%)

**Vascular territory**	
Anterior circulation	
MCA	23 (69.7)
Complete MCAS	4 (12.1)
Partial MCAS	19 (57.6)
ACA	4 (12.1)
Posterior circulation	
PCA	8 (24.2)
BA	3 (9.1)
SCA	2 (6.1)
AICA	1 (3.0)
PICA	8 (24.2)
Border zone	
Anterior	0 (0)
Posterior	5 (15.2)
Internal	3 (9.1)
**Lesion patterns**	
Single	
Pattern 1	5 (15.2)
Multiple	28 (84.8)
Pattern 2	11 (33.3)
Pattern 3	7 (21.2)
Pattern 4	10 (30.3)

MCAS, middle cerebral artery stroke; ACA, anterior cerebral artery; PCA, posterior cerebral artery; BA, basilar artery; SCA, superior cerebellar artery; AICA, anterior inferior cerebellar artery; PICA, posterior inferior cerebellar artery.

Of the 37 patients with a CVC, 25 were treated with surgical therapy. The mean duration from brain imaging to surgery was 42 days (range 3-143 days). Only 3 of 25 patients treated with surgery expired, while 7 out of 12 patients without surgery died during hospitalization (12.0% versus 58.3%, *P* = 0.006). Of the 25 patients treated with surgery, 4 underwent surgical treatment within 2 weeks after the diagnosis of a minor ischemic CVC (early surgery group), whereas the other 21 underwent surgical treatment after more than 2 weeks (delayed surgery group). Two of 4 patients who underwent early surgery had postoperative intracranial bleeding (left frontal SAH and intraventricular hemorrhage, respectively), whereas only one of 21 patients who underwent delayed surgery developed SAH and subdural hematoma in the right frontotemporal region postoperatively (50% versus 4.8%, *P* = 0.057). Among the 4 patients who underwent early surgery, the patient who developed intraventricular hemorrhage died from hydrocephalus and brain herniation, while 2 of 21 patients who underwent delayed surgery expired due to postoperative brain complications (the patient mentioned earlier) and septic shock, respectively (25.0% versus 9.5%, *P* = 0.422). Thus, the early surgery group had a statistical trend toward a higher risk of postoperative brain complications.

### Influence of a CVC on the outcome in patients with IE

The median follow-up period was 51.9 months (range 2 - 149 months). Of the 144 patients with IE, 28 died during hospitalization. The most common cause of in-hospital death was sepsis with multiple organ failure (18 cases, 64.3%), followed by postoperative ICH (3 cases, 10.7%), cerebral infarct progression (2 cases, 7.1%), preoperative ICH (2 cases, 7.1%), sudden cardiac arrest (2 cases, 7.1%) and postoperative bleeding complication (1 case, 3.6%). In addition, two patients expired due to pulmonary embolism and sudden cardiac arrest, respectively after hospital discharge. Of the 144 patients, 4 without a CVC and 2 with a minor CVC (4.2%) were lost during the follow-up period. These patients were censored at their last clinic visit.

**Figure 2:**
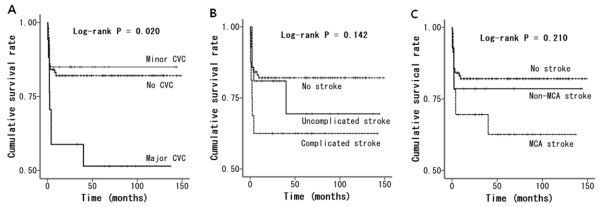
**Long-term survival rate according to the type of CVC.** CVC, cerebrovascular complication; MCA, middle cerebral artery.

During the follow-up period, the CVC group had a statistical trend toward a higher mortality, but did not show a significantly different risk of death from that in the no-CVC group (*P* = 0.141; HR 1.747; CI 0.831-3.673). When CVCs were classified according to the severity of initial neurologic symptoms, 20 patients and 17 patients belonged to the minor and major CVC groups, respectively. There was a significant difference in the survival rate among the no-CVC group, minor CVC group and major CVC group (Figure 2A, *P* = 0.020 by log rank test). The minor CVC group had a similar risk of death as the no-CVC group (*P* = 0.803; HR 0.856; CI 0.253-2.894), whereas the major CVC group had a significantly higher mortality (*P* = 0.013; HR 2.865; CI 1.254-6.548) than the no-CVC group.

Of the 20 patients with a minor CVC, 17 (85.0%) had no disability (mRS 0 or 1) and the other 3 (15.0%) were dead at 6 months. In contrast, only 2 (11.8%) of the 17 patients with a major CVC had no disability (mRS 0 or 1). In the remaining patients (88.2%), the outcome was disability or death at 6 months; 8 patients (47.1%) had a significant disability (mRS 2 or 3 in 7 patients and mRS 5 in 1 patient) and the other 7 (41.2%) were dead at 6 months.

Of the 37 patients with a CVC, 16 patients had a complicated stroke, which included 11 cases of ischemic stroke combined with hemorrhage, 1 case of ICH related to mycotic aneurysm, 1 case of ischemic stroke with meningitis, 2 cases of seizures associated with ischemic stroke and 1 case of ischemic stroke with ICH/seizure. There was no significant difference in mortality between the no-CVC group, uncomplicated stroke group and complicated stroke group (Figure 2B, *P* = 0.142). In addition, the vascular territory (the involvement of MCA territory) was not related to the risk of death in patients with IE (Figure 2C, *P* = 0.210).

As shown in Table 4, the univariate Cox regression model for mortality demonstrated that advanced age (≥ 65), diabetes mellitus, hypertension, atrial fibrillation, history of CHF, comorbidity index >2, prosthetic valve IE, S. aureus infection, no surgery and major CVC have a statistically significant impact on the risk of death.

**Table 4:** Results of the univariate and multivariate Cox proportional hazard models for mortality

	**Univariate**		**Multivariate (model 1)**		**Multivariate (model 2)**	
**Variables**	**HR (CI)**	***P***	**HR (CI)**	***P***	**HR (CI)**	***P***
Age ≥65	2.730 (1.297-5.748)	0.008	3.386 (1.563-7.335)	0.002	3.138 (1.421-6.930)	0.005
Male	1.534 (0.745-3.159)	0.245				
Diabetes mellitus	4.082 (1.975-8.435)	<0.001				
Hypertension	2.805 (1.311-6.005)	0.008				
Atrial fibrillation	2.152 (1.049-4.415)	0.037				
History of CHF	2.773 (1.354-5.679)	0.005				
Comorbidity index >2	6.002 (2.902-12.414)	<0.001				
Prosthetic valves	3.076 (1.438-6.578)	0.004			2.819 (1.315-6.044)	0.008
Mitral valve IE	2.189 (0.764-6.274)	0.145				
Aortic valve IE	0.719 (0.346-1.494)	0.377				
Dual valve IE	1.280 (0.570-2.875)	0.550				
Staphylococcus aureus	2.621 (1.166-5.891)	0.020				
No surgery	3.936 (1.895-8.177)	<0.001				
Major CVC	2.931 (1.304-6.586)	0.009	3.786 (1.635-8.771)	0.002	3.893 (1.649-9.194)	0.002

HR, hazard ratio; CI, 95% confidence interval; IE, infective endocarditis; CVC, cerebrovascular complication.

In addition, two levels of multivariate analysis were performed to evaluate the impact of a major CVC on mortality. In the model 1 where only the age was adjusted, major CVC (*P* = 0.002; HR 3.786; CI 1.635-8.771) was significantly associated with high risk of death in patients with IE. In the model 2, prosthetic valve IE was added as a variable in the multivariate analysis. Comorbidity and S. aureus were excluded despite their statistical significance in the univariate analysis because they were closely related to major CVC (*P* = 0.011 and 0.012 respectively by Chi-square test) thus having a potential to behave as redundant variables (multicollinearity). In this analysis, major CVC (*P* = 0.002; HR 3.893; CI 1.649-9.194) remained a significant predictor of mortality in IE patients, together with advanced age (*P* = 0.005; HR 3.138; CI 1.421-6.930) and prosthetic valve IE (*P* = 0.008; HR 2.819; CI 1.315-6.044).

## Discussion

A CVC was observed in 25.7% of the patients with IE, which is within the range of 10 to 80% that has been reported in previous studies [2,3,5,13-16]. A wide variation in the prevalence among studies may be due to the failure to detect mild or transient neurologic signs in critically ill patients leading to an underestimation of CVC, especially in retrospective studies that used chart reviews. In contrast, the rate of IE-related CVC can be increased more in patients with S. aureus infection and severe form of the disease necessitating intensive care unit admission [2,3]. Additionally, more frequent scanning of brain, especially using diffusion-weighted MRI, enhances the detection rate of brain lesions in IE patients. Several prospective studies with a higher frequency of brain imaging showed that the incidence of CVC could reach 65-80% although many patients were asymptomatic [15,16].

In many previous studies, S. aureus infection has been found to be associated with a high rate of stroke and mortality in IE patients [3,17,18]. Our results also showed that S. aureus infection has a significant relationship with the occurrence of a CVC and death on the univariate analysis. In addition, multivariate analysis performed in this study demonstrated that S. aureus infection together with mobile vegetation independently predicted CVC in IE patients.

The most common type of neurologic complication related to IE was acute ischemic stroke (30 cases, 20.8%), followed by hemorrhagic stroke (16 cases, 11.1%), seizures (3 cases, 2.1%) and TIA (2 cases, 1.4%). SCE, meningitis and mycotic aneurysm were found in only l patient (0.7%), each. Of these, SCE and meningitis had a lower incidence rate (0.7%) in our study than that in previous recent studies, in which SCE was found in 4-30% of patients with IE [3,5,16] and meningitis was found in 3-25% of patients with IE [1,4,19]. This could be explained partly by the non-routine use of brain imaging in this study. For example, most of the brain scans seem to have been done only when the patients had a clinical symptom suggesting brain complications. In addition, headache, if mild, may not have been investigated for meningitis in patients with serious heart conditions.

Acute ischemic lesions in IE patients are mostly multiple, and mainly located in the MCA territory corresponding with the cardioembolic stroke subtype. In addition, various patterns of lesions including single, territorial, disseminated punctate, and disseminated small and large lesions were seen. These results of this study coincide with those of a previous study [12], which showed that IE-related strokes had all the patterns of lesions from pattern 1 to 4, whereas strokes associated with adenocarcinoma-related, nonbacterial thrombotic endocarditis (NBTE) had only pattern 4 lesions. This difference in lesion patterns between two diseases can be explained by the presumption that vegetation in IE causes an inflammatory reaction within itself, and hence the vegetation can conglomerate more firmly and will not fragment more easily than that in NBTE.

During cardiac surgery, neurological deterioration can occur in patients with IE and CVC. This is presumed to be attributable to intralesional hemorrhage related to heparinization and extension of infarcted area resulting from hypotension during cardiopulmonary bypass [20]. Previous studies have shown that the risk of neurologic deterioration related to cardiac surgery declines over the first month [21,22]. Therefore, the recent guideline of the Society of Thoracic Surgeons recommends delaying the surgery for at least 4 weeks from the stroke onset in IE patients with a major ischemic stroke or any intracranial hemorrhage, and permits a shorter delay of 2-4 weeks in the patients with a small brain infarct and severe cardiac conditions necessitating urgent surgery [22]. Our study also showed that early surgery within 2 weeks seemed to increase the probability of neurological deterioration, although this result did not reach statistical significance due to the small sample size.

However, several recent studies reported that the risk of neurological deterioration due to early cardiac surgery may be lower than that assumed previously, except in patients with intracranial bleeding [23,24]. They emphasized that early cardiac surgery is not contraindicated after ischemic stroke and can be performed without delay when there are indications for surgery. Thus, the optimal timing of cardiac surgery in IE patients with a CVC is controversial, and large-scale controlled studies are required in the future. Accordingly, the decision regarding the timing of surgery should be individualized, and based on a multidisciplinary approach to optimize the combined management of the cardiac and cerebral diseases.

In this study, total CVC was not significantly related to higher mortality in IE patients. Instead, the risk of death differed according to the severity of the initial neurologic symptom of CVC. Patients with a minor CVC had a better prognosis than patients with a major CVC, and there was no difference in mortality between the minor and no CVC groups. In contrast, major CVC proved to be a significant determinant of mortality in IE patients. This result is similar to that of a previous study, which demonstrated that high risk of death in IE patients was related to clinically overt stroke, but not to SCE or TIA [5].

Previous studies have discussed the association between CVC and long-term outcome in patients with IE [2-5]. Some previous studies showed similar results to those in our study, thereby indicating that total CVC was not significantly associated with the risk of death in IE patients [4]. However, other studies reported that neurologic complication was an independent predictor of mortality in IE patients [2,3]. This might be attributable to the difference in the study population, management protocol like the timing of cardiac surgery or statistical variables between the studies.

The present study has several limitations. First of all, the main limitation of this study may be the small sample size based on a single-center experience resulting in the attenuation of statistical power in the mortality analysis. Second, patients were not managed according to a standardized protocol, and the data were collected retrospectively. Thus, an arbitrary decision about patient management could have influenced the results of this study. For example, brain imaging was performed in only about half of the study patients based on the decision of the attending physician in each situation. Particularly, the critically ill patients did not seem to undergo MRI because it could put them at a greater risk. Consequently, the rate of asymptomatic brain infarct could have been underestimated in comparison with that in the results of several previous studies.

## Conclusions

IE can give rise to various forms of CVC, most frequently, acute ischemic brain lesions. In our study, major CVC (initial mRS ≥ 3) was associated with high risk of mortality although total CVC was not significantly related to the risk of death in patients with IE.

## Competing interests

The authors declare that they have no competing interests.

## Authors’ contributions

All authors met the criteria for authorship and have approved the contents of the text. SJL and DSL contributed to study concept and design. SJL did statistical analysis, wrote the first draft and revised the manuscript. SSO, DSL, CYN and JHK participated in the acquisition, analysis and interpretation of data.

## Pre-publication history

The pre-publication history for this paper can be accessed here:

http://www.biomedcentral.com/1471-2377/14/30/prepub
